# Use of Neuroenhancement Drugs: Prevalence, Frequency and Use Expectations in Switzerland

**DOI:** 10.3390/ijerph110303032

**Published:** 2014-03-12

**Authors:** Stéphane Deline, Stéphanie Baggio, Joseph Studer, Alexandra A. N’Goran, Marc Dupuis, Yves Henchoz, Meichun Mohler-Kuo, Jean-Bernard Daeppen, Gerhard Gmel

**Affiliations:** 1 Alcohol Treatment Centre, Lausanne University Hospital (CHUV), Av. Beaumont 21 Bis, Pavillon 2, Lausanne CH-1011, Switzerland; E-Mails: Joseph.Studer@chuv.ch (J.S.); Adjua-Alexandra.NGoran@chuv.ch (A.A.N.); Yves.Henchoz@chuv.ch (Y.H.); Jean-Bernard.Daeppen@chuv.ch (J.-B.D.); Gerhard.Gmel@chuv.ch (G.G.); 2 Institute for Social Sciences, University of Lausanne, Geopolis Building, Lausanne CH-1015, Switzerland; E-Mail: stephanie.baggio@unil.ch; 3 Institute of Psychology, University of Lausanne, Geopolis Building, Lausanne CH-1015, Switzerland; E-Mail: Marc.Dupuis@unil.ch; 4 Institute of Social and Preventive Medicine, University of Zurich, Hirschengraben 84, Zurich CH-8001, Switzerland; E-Mail: meichun.mohler-kuo@uzh.ch; 5 Addiction Switzerland, Case Postale 870, Lausanne CH-1001, Switzerland; 6 Centre for Addiction and Mental Health, 250 College St., Toronto ON M5T 1R8, Canada; 7 University of the West of England, Frenchay Campus, Coldharbour Lane, Bristol BS16 1QY, UK

**Keywords:** college students, expectations of use, neuroenhancement, prevalence, smart drugs

## Abstract

*Objective*: The present study investigates the use expectations, prevalence and frequency of neuroenhancement drug (ND) use among the Swiss male population, separating college students from others. *Methods:* Young Swiss men were invited to participate in the Cohort Study on Substance Use Risk Factors. A total of 5,967 participants responded to questions on six types of NDs (wakefulness medication, antidepressants, Alzheimer’s disease medication, Parkinson’s disease medication, attention deficit-hyperactivity disorder (ADHD) medication, and beta-blockers). The frequency of use depending on five expectations (to enhance wakefulness, attention, memory, concentration and stress reduction) was analyzed for a twelve-month period. *Results:* (1) About 3% of the sample indicated use of at least one ND; (2) ADHD medication was the most prevalent; (3) The type of ND preferred differed depending on academic status (4). Quantitatively, over the year, college student users used ND much less frequently than other users. *Conclusions:* Prevalence of ND use is low in Switzerland relative to other countries such as the United States. Patterns of ND use differed depending on academic status, suggesting that while college student ND users tended to do so rarely (probably to enhance cognitive abilities for exams), non-college male users used other NDs more frequently (probably to “get high”).

## 1. Introduction

Psychopharmacological neuroenhancement refers essentially to the use of medication as a potential “cognitive enhancer” in order to better perform in studies or at work [1], or to regulate mood, stress or anxiety [2], which could indirectly enhance cognitive performance [3]. Stimulants, such as methylphenidate (better known as Ritalin^®^), modafinil, selegiline and donepezil, as well as some tranquilizers such as beta-blockers or anxiolytics, are commonly prescribed as medication for particular diseases (attention deficit-hyperactivity disorder (ADHD), narcolepsy, Parkinson’s disease, Alzheimer’s disease, heart disease, anxiety disorders), but are then often used without a doctor’s prescription, or for reasons other than prescribed [4]. 

The increase in the prevalence of neuroenhancement drug (ND) use was described more than a decade ago [5]. Most studies in the field of neuroenhancement have taken place in the USA [6,7,8,9] or in Canada [10], and only a small number have been initiated in other countries (most notably in Australia [11] or Europe [2,12,13,14,15]). Although the twelve-month prevalence of ND use among young people (between 18 and 24–25 years old) appeared to be high in the US (e.g., 4.3% for ADHD medication [16], 0.3% for Provigil^®^ [5]), elevated prevalence rates have also been found in the general population (e.g., 1.5% for non-prescribed anxiety medication [17]). However, little is known about cases in other countries. In an effort to compensate for this lack of information, this study investigates ND use in a broad sample of the young Swiss male population.

Numerous studies about ND use have been more specifically focused on college student use in the US (e.g., [8,9]). NDs, particularly ADHD medication, have become the second most frequently used type of illicit drugs among college students after cannabis [18] with, notably, a prevalence over the past year of 2.3% for Ritalin^®^ and 9.8% for Adderall^®^ [5]. A recent review also indicated that prevalence over the past year, with regard to the ADHD medication, has generally oscillated around 5%–9% among college students in the USA [19]. A similarly high rate of 6% has been found in Canadian college students [10]. Little knowledge exists for other parts of the world and it would seem that in Europe at least, neuroenhancement, or more specifically the use of “cognitive enhancers”, is rather low (e.g., 4.3% in Belgium [15], 4.1% in Switzerland [2] and between 1%–3% in Germany [12,14]). One explanation for such higher prevalence among US college students is the academic pressure [20]. Confronted with a highly competitive academic context, college students use NDs in order to increase cognitive capacity during exams [21] or to regulate mood, stress or anxiety [2] which could indirectly increase cognitive performance [3]. Moreover, such behavior is encouraged by students’ over-estimation of how many of their peers use NDs [22], over-prescription of NDs by physicians [23], and by their erroneously-perceived safety, regardless of the potential risk for abuse and dependency [20,24,25]. While several reasons for use (*i.e.*, “increased attention”, “increased memory capacity”, “staying awake” or “getting high”) have been reported in previous studies [7,9,13,20], information on use by groups other than US and college student populations is still lacking with regard to the different types of NDs. In an effort to clarify the specificity of college student ND use, this article investigates frequency of use and ND use expectations for the different types of ND, depending on academic status.

The purposes of this study were thus threefold: (1) to estimate the prevalence of ND use among young adults in a broad sample of men in Switzerland; (2) to explore ND use expectations and their related frequency of use; and (3) to study the association between academic status and use of NDs. It was hypothesized that the prevalence and regularity of ND use differed between male college students and other young men.

## 2. Methods

### 2.1. Data Collection

Attendance at a military service drafting day is mandatory for all young Swiss males of around 20 years of age. Since there is no pre-selection procedure these days provide a virtual census of the corresponding Swiss male population. Although women can serve in the Swiss army, this is on a voluntary basis and therefore women showing up in conscription centers were not representative of women in Switzerland of this age group and were not eligible for inclusion. The data used in the present study come from participants enrolled at three of Switzerland’s six recruitment centers as part of the longitudinal Cohort Study on Substance Use Risk Factors (C-SURF). Between August 2010 and November 2011, potential recruits were invited to participate in this longitudinal study and each participant received a voucher worth CHF 30 ($30, €25) upon completion of the questionnaire. 

A total of 7,563 individuals gave written consent to their participation and 5,990 (79.2%) actually filled out the baseline questionnaire (which was approximately 45–60 min in length, up to 153 questions among which 92 questions were on substance use) that was sent to their home address about two weeks later. Responses were thus completely independent of recruitment into the army and confidentiality was ensured because military authorities were not informed about the responses to the questionnaire. Non-response bias was low (for more details on the sample, see [26]). Missing and aberrant values for either of the two neuroenhancement questions (use expectations and type of medication; see below) were excluded (n = 23), resulting in a final sample size of 5,967 participants for the present study (99.6% of the initial sample). The study protocol was approved by Lausanne University Medical School’s Clinical Research Ethics Committee ([27]).

### 2.2. Measurements

#### 2.2.1. Use Expectations and ND Use

The questionnaire described ND use as medication usually prescribed for specific diseases, but used for other reasons, e.g., with the expectation of increased concentration, learning capacities, attention or memory spans, as well as decreased stress during exams, or to give oneself the impression that one is performing better. ND use was assessed using two questions. Participants first indicated how often they had used NDs over the past 12 months for each of the five following expectations: (a) to raise vigilance capacity, effectiveness or energy; (b) to increase capacity to pay attention or to concentrate at work; (c) to raise the capacity to remember things in general, as well as to learn and recall matters; (d) to improve concentration and cognitive capacities; or e) to reduce anxiety or stress, e.g., during exams. Thus, here, expectation is used as the reason for self-medication using NDs. The first four expectations were related to cognitive enhancement, while the last was linked to stress and tension reduction. For each expectation, answers were collected on an 8-point scale: “Never” (coded 0); “Once” (coded 1); “2–3 times a year” (coded 2.5); “4–9 times a year” (coded 6.5); “Once or twice a month” (coded 18); “3–4 times a month (coded 42)”; “2–3 times a week” (coded 130); and “4 times a week or more” (coded 286). Explicitly providing the use expectations allows ND use memories to be primed, and also makes it easier to measure the annual frequencies of ND use. Prevalence over the past 12 months was obtained by dichotomizing the annual frequencies (“Never” *vs.* “At least once” during the past 12 months). Secondly, those participants who had indicated at least one expectation for ND use during the past 12 months were asked which types of NDs they had used. Six types of ND were suggested, with examples of active ingredients and the most common brands being given: (a) wakefulness medicaments for such sleeping disorders as narcolepsy e.g., modafinil (Provigil^®^) and armodafinil (Nuvigil^®^); (b) antidepressants, e.g., venlafaxine (Effexor^®^) and fluoxetine (Prozac^®^); (c) Alzheimer’s disease drugs, e.g., donepezil (Aricept^®^); (d) Parkinson’s disease drugs, e.g., selegiline (Jumexal^®^); (e) drugs for ADHD, e.g., methylphenidate (Ritalin^®^); (f) beta-blockers for cardiac troubles, e.g., atenolol (Tenormin^®^). An additional open-ended “Other” category was provided, enabling participants to name other substances ingested. This is a common procedure in health surveys where it is difficult to provide respondents with a comprehensive list of medication. Questions therefore initially focus on potential indications (e.g., medication to relieve pain or to facilitate sleep).

Analysis of the 73 responses to the “Other” category showed that 51 did not remember the substance used, or referred to substances or medication that could not be considered as NDs (e.g., homeopathy, creatine or dextrose). Thus only the remaining 22 cases with valid responses only (e.g., caffeine tablets, Tonoglutal^®^ or Adderall^®^) were coded, in accordance with the six aforementioned ND types. Multiple responses were possible. Single users were defined as those using only one of the six types of ND; poly-users were those using more than one type of ND. Only individuals who indicated at least one expectation for ND use, and had used one valid ND type over the past 12 months, were analyzed as ND users.

#### 2.2.2. Academic Status

In order to measure whether NDs were used differently by college students and non-college men, their current activity was assessed by requesting their “current educational or work status” (e.g., “Basic vocational education”, “University”, “Paid job”). One variable, “Academic status”, was created by roughly coding the USA “College student status”. This status was assigned when the current educational status corresponded to attendance at a school issuing education beyond that of schools with a formal 13-year education system. Non-college men referred to all others, including those still in school or currently in a paid job. 

#### 2.2.3. ADHD Criteria

ADHD criteria were assessed using the Adult ADHD Self-Report Scale [28], a 6-item questionnaire for screening attention deficit-hyperactivity (α = 0.80). Each question—associated with feelings or behaviors—was answered on a five-point Likert frequency scale, ranging from “Never” (coded 0) to “Very often” (coded 4). A summary score was generated ranging from 0 to 24, a positive ADHD screen being defined as a score equal to or greater than 14 [29]. This score was made in order to control for the potential use of ADHD medication as a ‘prescribed’ medicament for ADHD.

### 2.3. Statistical Analyses

First of all, descriptive statistics provided information about ND prevalence, use expectations and frequency of use. Then Chi-square analyses were used to test firstly the hypothesis of a difference in ND use prevalence between college students and non-college men and secondly the association between the use of ADHD medication and a diagnosis of ADHD. This analysis was done to test whether ADHD medication may actually have been prescribed for this medical condition, because the present study was designed to focus on the non-prescribed use of NDs. Finally, logistic regressions among ND users were performed in order to test how the use of each type of ND could be predicted by academic status. Analyses were performed using the SPSS 21 software. 

## 3. Results

### 3.1. Prevalence of ND Uses and Use Expectations

Table 1 shows the past year prevalence rate (PYPR) of ND use and the ND use expectations of the young male population in Switzerland. A total of 180 participants (3.0% of the total sample) indicated at least one expectation for taking NDs at least once during the past 12 months, and indicated at least one ND type. The most common ND use expectations were to increase wakefulness, energy and productivity (64.4%), and to increase concentration and other cognitive abilities (59.4%). The least common ND use expectation was to reduce anxiety or stress (43.9%). Most ND users (92.2%, n = 166) used only one type of ND. The most prevalent ND among single users was ADHD medication (61.5%). The next most used ND types were wakefulness medication (13.9%), antidepressants (10.8%), beta-blockers (10.2%), Parkinson’s disease drugs (2.4%) and Alzheimer’s disease drugs (1.2%). Among “poly-users” (n = 14), 12 used ADHD medication in addition to other NDs; one user took wakefulness and Alzheimer’s disease drugs, and another took antidepressants and Parkinson’s disease drugs. 

**Table 1:** Prevalence rates of neuroenhancement drug use, and neuroenhancement use expectations among users.

N = 5,967		n	% of Total Sample	% of ND Users	% of Single Users
**Neuroenhancement Drug (ND) Users**		**180**	**3.02**	-	-
Single Users	Wakefulness medication	23	0.39	12.8	13.9
	Antidepressants	18	0.30	10.0	10.8
	Alzheimer’s disease medication	2	0.03	1.1	1.2
	Parkinson’s disease medication	4	0.07	2.2	2.4
	ADHD medication	102	1.71	56.7	61.5
	Beta-blockers (cardiac)	17	0.28	9.4	10.2
Poly-users		14	0.23	7.8	-
**Expectations Associated with ND Use**					
Increase wakefulness, energy, productivity		116	1.94	64.4	-
Increase attention span, extend memory load		89	1.49	49.4	-
Increase memory and learning capacity		89	1.49	49.4	-
Increase concentration and other cognitive abilities		107	1.79	59.4	-
Reduce anxiety or stress (e.g., during exams)		79	1.32	43.9	-

### 3.2. Expectations and Frequency of ND Use

In order to study the relationship between the expectations of use and the types of ND, and due to the small sample quantities of users, ND types were pooled into 3 main categories, according to the main *a priori* “expected” effect of each medication on emotion regulation or cognitive function. Firstly, we grouped together the beta-blockers and the antidepressants expected to act as tranquilizers; these medications being used for anxiety disorder [30], this user category was labeled the “anti-anxiety medications” group (n = 35; PYPR = 0.59%).

Secondly, we grouped users and poly-users of at least one kind of ADHD medication, into a category labeled “prescription stimulant medication” (PSM) (n = 114; PYPR = 1.91%), as the non- medical use of ADHD medication is called in the literature [31]. ADHD medication was specifically expected to affect attentional processes (e.g., attentional control) and can increase attentional cognitive efficiency (e.g., attentional focus), but it also disturbs other attentional mechanisms (e.g., focus switching), which could decrease cognitive capacity (e.g., learning). 

Thirdly, wakefulness drugs, Alzheimer's disease or Parkinson’s disease medication were expected to enhance cognitive ability such as memory and learning mechanisms; their users were put into the category labeled “cognitive enhancers” (n = 30; PYPR = 0.50%). Wakefulness drugs were expected to increase cognitive capacity by helping students to stay awake and thus study longer. One case involving a dual user of an antidepressant and a Parkinson’s disease drug was excluded from further analysis. 

The total sample contained 11.5% (n = 684) college students and 88.5% (n = 5,283) non-college men. Among ND users, 14.5% (n = 26) were college students and 85.5% (n = 153) were non-college men. There was no significant difference in prevalence of any ND use (all types taken together) between college student users (3.80% of college students) and non-college users (2.90% of non-college men) (χ²(1, N = 5,967) = 1.71, *p* = 0.19). The logistic regression examining the association between the type of ND used (cognitive enhancers, anti-anxiety medications and PSM) and academic status showed that the prevalence of ND type differed between college students and non-college men (Table 2). The prevalence of cognitive enhancer use among college student ND users was greater than among non-college men ND users (OR = 2.65 (1.03–6.83)). On the contrary, the prevalence of PSM use among college student ND users was less than among non-college ND users (OR = 0.43 (0.18–0.99)). There was no significant difference in the use of anti-anxiety medications between these groups. A positive screening for ADHD –carried out by using a cut-off criteria– was not significantly associated with the use of PSM among ND users (χ²(1, n = 179) = 0.22; *p* = 0.640), which shows that the aim of finding non-medical prescription drug users was achieved. 

**Table 2:** Associations between neuroenhancement drug use and academic status.

Income	Neuroenhancement Drugs	Odds Ratio	R-square	95% CI		Sig.
Academic status (reference: non-college men)	Cognitive enhancer	2.65	0.035	1.03	6.83	0.044
	Anti-anxiety medication	1.28	0.002	0.47	3.48	0.625
	Prescription stimulant medication	0.43	0.029	0.18	0.99	0.048

The expectations and frequencies of use for each ND type according to academic status are shown in Table 3. NDs were used in accordance with their respective main pharmacological effect among healthy people. Firstly, for ND users from both academic groups, the most expected effect of anti-anxiety medication use was reduced anxiety or stress (83.3% for the college student user group and 72.4% for non-college men users). Secondly, both college student and non-college men users of PSM had diverse cognitive expectations, since at least half of these users (*i.e.*, between 50% and 75% of them) endorsed the first four expectations, *i.e.*, all expectations not related to stress reduction. Finally, cognitive enhancers were also rarely used in order to reduce stress and anxiety. However, they were used for different enhancement expectations depending of academic status: college student users more often indicated an expectation of an increase in memory (87.5%) and attention span (75.0%), whereas non-college men expected to increase wakefulness (68.2%) and concentration (72.7%).

**Table 3:** Proportion of neuroenhancement drug users and annual frequency of use depending on expectation and academic status.

Neuroenhancement Drug	Cognitive Enhancer (n = 30)			Anti-anxiety Medication (n = 35)			Prescription Stimulant Medication (n = 114)		
College students: n = 26 (14.5% of users)	n = 8 (26.7%)			n = 6 (17.1%)			n = 12 (10.5%)		
Expectations	%	M	SD	%	M	SD	%	M	SD
Wakefulness, energy	62.5	3.8	2.5	16.7	1.0	0.0	58.3	5.2	6.2
Attention span	75.0	3.1	2.7	16.7	1.0	0.0	75.0	4.4	5.6
Memory/learning	87.5	2.8	2.6	33.3	1.0	0.0	50.0	5.3	6.6
Concentration	62.5	3.5	2.8	50.0	2.8	3.2	66.7	4.2	5.9
Anxiety or stress	37.5	3.3	2.8	83.3	1.6	0.8	33.3	13.0	19.5
Non-college men: n = 153 (85.5% of users)	n = 22 (73.3%)			n = 29 (82.9%)			n = 102 (89.5%)		
Expectations	%	M	SD	%	M	SD	%	M	SD
Wakefulness, energy	68.2	33.0	77.7	41.4	50.5	88.8	73.5	40.0	89.5
Attention span	40.9	7.0	13.2	37.9	39.2	90.5	51.0	57.0	103.1
Memory/learning	45.5	9.1	13.4	41.4	60.1	111.7	50.0	51.6	99.2
Concentration	72.7	23.3	70.9	48.3	51.4	105.1	58.8	53.3	101.5
Anxiety or stress	45.5	4.0	5.2	72.4	31.0	85.2	34.3	32.8	82.0

Notes: % = percent of neuroenhancement drug (ND) users per subgroup (cognitive enhancers, anti-anxiety medications; prescription stimulant medication) according to academic status, indicating the corresponding expectation. M = mean annual frequency of ND use for each expectation, among users for the indicated expectation. SD = Standard deviation. Remark: One antidepressant-Parkinson’s disease drug dual-user was excluded.

When considering the most expected effect mentioned for each ND, the two academic groups diverged in terms of the number of times those NDs were used per year. For each of the three ND categories, the subgroup of college students who declared the most popular expectation (*i.e.*, 75.0% of PSM users mentioned attention; 83.3% of anti-anxiety medication users mentioned anxiety and stress reduction; and 87.5% of cognitive enhancer users mentioned memory and learning) tended to use those NDs fewer than five times a year (4.4, 1.6 and 2.8 times a year, respectively). 

For each of the three ND categories, the subgroup of non-college men users who declared the most popular expectation, (*i.e.*, 73.5% of PSM users mentioned wakefulness and energy; 72.4% of anti-anxiety medication users mentioned anxiety and stress; and 72.7% of cognitive enhancer users mentioned concentration) tended to use those NDs much more frequently than college students, *i.e.*, approximately at least twice a month (40.0, 31.0 and 23.3 times a year, respectively). In general, non-college men used NDs more frequently than college student users, and this was true with respect to all the expectations.

## 4. Discussion

This study investigated the prevalence of ND use and ND use expectations among the young Swiss male population. It supplements the knowledge stemming from existing US studies, based mostly on college students, by comparing college student ND users with non-college men ND users of the same age. The overall prevalence rate of potential ND use in this study was relatively low (3.0%). The most popular type of ND was ADHD medication, used by 61.5% of single-type ND users (and 12 out of 14 poly-users). Wakefulness medication, antidepressants and beta-blockers come after ADHD medication, with around 10%–13% of ND users. This confirms USA literature insofar as ADHD medication (*i.e.*, Ritalin^®^, Adderall^®^ and Concerta^®^) is the most prevalent type of ND used among young Swiss men, though their PYPR only corresponds to 1.91%. However, this was much lower than in most US studies, where they range from 5%–9% [19]. Our results were more in line with studies conducted in Germany (1%–3%) [12,14]. Similarly, the ND use of ‘anti-anxiety medications’ (PYPR = 0.59%) was approximately three times less than in USA studies [17]. Only “cognitive enhancers”, such as wakefulness medication (e.g., Provigil^®^), seemed to be used on a similarly rare basis (PYPR = 0.50%) to the USA [5]. 

The ND expectations essentially sought for were increased wakefulness (1.94% of the total sample, 64.4% of ND users), concentration or other cognitive capabilities (1.79% of the total sample, 59.4% of ND users), as stated in previous studies [1,9]. The association between ND use and the use expectations was investigated by verifying which expectation of ND use was mentioned as sought after by participants. In general, NDs were used in accordance with their pharmacological effects: “anti-anxiety medications” were mainly used to reduce stress and anxiety, while cognitive enhancers or PSM—including ADHD medication—were used to stay awake longer and to increase the overall cognitive abilities. Hence, the results indicated that NDs were indeed used for the specific effects ascribed *a priori* to each medication, *i.e.*, the same effect on cognition for both healthy and ill people. It should be noted, however, that the effects of cognitive enhancers and PSMs on healthy people, are still scientifically unclear and debated [1,32,33,34].

Using logistic regressions, college student users were more likely to use cognitive enhancers (though not ADHD stimulants) than non-college men users. This finding shows that the college students in our sample probably used cognitive enhancers more often in order to increase attention span, memory and learning capacities, as recorded in the literature [24], while non-college men used them to stay awake and concentrated. On the other hand, whereas PSMs are generally observed to be highly prevalent among college students in the USA [5], it was non-college men who were more likely to use PSMs in Switzerland. Moreover, we also tested for ADHD symptomatology; the results revealed that the use of PSMs was clearly unrelated to ADHD symptomatology, and they were clearly being used as non-medically prescribed drugs. 

Analyses of the ND use expectations and of the frequency of ND use provided additional important indications about the specificity of use of both academic groups. Among users, the college student group used all different types of NDs fewer times per year than the users in the non-college men group. For example, college student users of PSM used it nearly 9 times less frequently (75% of them used PSM 4.4 times per year on average) than non-college men users (73.5% of them used PSM 40.0 times per year on average). These findings, combined with the difference in the proportions of types of NDs used depending on academic status, illustrates that the misuse of NDs depended on the target of use: college students probably used more cognitive enhancers than PSMs (commonly containing amphetamines) for academic performance, and therefore more parsimoniously, *i.e.*, a few times per year at most, in order to increase cognitive abilities during exams [35]. Non-college men used more PSM (and therefore the amphetamine stimulants) than cognitive enhancers, and far more frequently; *i.e.*, at least a few times per month; e.g., possibly to stimulate energy and to “get high” [9,36], regardless of the risk for abuse and potential toxicity [37]. If this assumption were true, this study’s findings also show the potential for misuse of NDs among young adult males in Switzerland. Whereas, college students used NDs rarely, others users tended to use NDs on a near weekly basis, probably without knowing their potential undesirable effects. These findings have revealed the necessity to study the effects of those drugs on cognitive function and to develop preventive strategies targeting the risks involved. As such, it might be useful for future research to focus on the regularity and amount of NDs used by college students, but also by the general population as a high amount of use multiplies the potential health risks [34,37]. 

Also somewhat surprising, however, was the difference in the prevalence of ADHD medication between Swiss and US college students. One assumption is that young adults use ND drugs because they are available. Hence, the prevalence of ADHD medication among US college students could be linked to the high accessibility of ADHD medication among students [35] (itself linked to the high rate of ADHD prescription in the US, e.g., [38]) and a diversion mechanism with regard to prescribed medications between peers [10,39]. In parallel, recent studies in Europe have indicated an increase in the number of prescriptions for methylphenidate [40,41], which may increase its accessibility and thus its prevalence rate. Monitoring the evolution of ND use in Europe is therefore warranted. 

## 5. Limitations

Our study nevertheless had its limitations. The first was the all-male sample itself. Additional studies in Switzerland should consider whether the results differ between men and women as previous studies having shown gender differences for both the expectations of use or ND use itself [7,36]. A second limitation was the limited and predefined expectations of use, which could exclude some ND users (e.g., recreational users). Another limitation was that the differentiation between the various types of NDs is sometimes ambiguous, with some responses in the open-ended “Other” category (e.g., homeopathics or vitamins) showing that young men of this age were not yet very familiar with the concept of NDs. This was also backed up by the fact that the most common responses in the “Other” category were “I don’t know what I took”, and “I don’t remember the name”. Additional categorical information on the different ND brands and names should also be provided in order to avoid such responses. Another methodological limitation of this study was that due to the limited quantity of users for certain types of ND, subgroups had to be made in order to summarize data, *e.g.* the PSM subgroup included a few users of drugs from more than one category, which could in some cases cause misinterpretation. Finally, the limited number of users meant that the significant logistical results should be taken with caution as a small change in the number of users could change the result.

## 6. Conclusions

The present study features several notable strong points on neuroenhancement drug (ND) use in the young Swiss male population. Despite lower prevalence rates than in the US, ND use, and particularly the non-medical prescription drug use of ADHD medication, could quickly increase. This could become a health problem, particularly as many of the effects of NDs may not actually be beneficial for cognitive performance at all [1], sometimes even posing risks for abuse or potential dependency [17]. The present study also shows that ND use by college students may not be representative of general ND use. It revealed that college students use NDs as cognitive enhancers relatively parsimoniously, possibly to enhance their cognitive abilities for exams; whereas non-college men use more prescription stimulant medication and far more frequently per year, possibly to stay awake longer at parties, or to “get high”. 
